# Bioinformatics analysis of miRNA and mRNA expression profiles to reveal the key miRNAs and genes in osteoarthritis

**DOI:** 10.1186/s13018-021-02201-2

**Published:** 2021-01-19

**Authors:** Pei-yan Huang, Jun-guo Wu, Jun Gu, Tie-qi Zhang, Ling-feng Li, Si-qun Wang, Minghai Wang

**Affiliations:** Department of Orthopaedic Surgery, Shanghai Fifth People’s Hospital Affiliated to Fudan University, No. 128 Ruili Road, Minhang District, Shanghai, 200240 China

**Keywords:** Osteoarthritis, Differentially expressed genes, Differentially expressed miRNAs, Transcription factors, Regulatory network

## Abstract

**Background:**

Osteoarthritis (OA) is a chronic degenerative joint disease and the most frequent type of arthritis. This study aimed to identify the key miRNAs and genes associated with OA progression.

**Methods:**

The GSE105027 (microRNA [miRNA/miR] expression profile; 12 OA samples and 12 normal samples) and GSE48556 (messenger RNA [mRNA] expression profile; 106 OA samples and 33 normal samples) datasets were selected from the Gene Expression Omnibus database. Differentially expressed genes (DEGs) and miRNAs (DEMs) were analyzed using the limma and ROCR packages in R, respectively. The target genes that negatively correlated with the DEMs were predicted, followed by functional enrichment analysis and construction of the miRNA-gene and miRNA-transcription factor (TF)-gene regulatory networks. Additionally, key miRNAs and genes were screened, and their expression levels were verified by real-time quantitative reverse transcription polymerase chain reaction (qRT-PCR).

**Results:**

A total of 1696 DEGs (739 upregulated and 957 downregulated) and 108 DEMs (56 upregulated and 52 downregulated) were identified in the OA samples. Furthermore, 56 target genes that negatively correlated with the DEMs were predicted and found to be enriched in three functional terms (e.g., positive regulation of intracellular protein transport) and three pathways (e.g., human cytomegalovirus infection). In addition, three key miRNAs (*miR-98-5p*, *miR-7-5p*, and *miR-182-5p*) and six key genes (murine double minute 2, *MDM2*; glycogen synthase kinase 3-beta, *GSK3B*; transmembrane P24-trafficking protein 10, *TMED10*; DDB1 and CUL4-associated factor 12, *DCAF12*; caspase 3, *CASP3*; and ring finger protein 44, *RNF44*) were screened, among which the *miR-7-5p → TMED10/DCAF12*, *miR-98-5p → CASP3/RNF44*, and *miR-182-5p → GSK3B* pairs were observed in the regulatory network. Moreover, the expression levels of *TMED10*, *miR-7-5p*, *CASP3*, *miR-98-5p*, *GSK3B*, and *miR-182-5p* showed a negative correlation with qRT-PCR verification.

**Conclusion:**

*MiR-98-5p*, *miR-7-5p*, *miR-182-5p*, *MDM2*, *GSK3B*, *TMED10*, *DCAF12*, *CASP3*, and *RNF44* may play critical roles in OA progression.

**Supplementary Information:**

The online version contains supplementary material available at 10.1186/s13018-021-02201-2.

## Background

As a chronic degenerative joint disease, osteoarthritis (OA) is induced by the destruction of articular cartilage and bone tissue [1]. The most common symptoms of OA include joint pain, joint swelling, stiffness, and limited motion range [2]. OA, which usually occurs in the lower back, fingers, hips, neck, and knees, is mainly caused by abnormal development of the affected limb or joint, joint injury, and genetic factors [3, 4]. At present, the therapeutic options for OA include reduction of joint stress, exercise, pain medications, and support groups [5]. OA is the most frequent type of arthritis and affects approximately 237 million people globally [6]. Therefore, exploring the mechanisms of OA is necessary in order to improve its diagnosis and therapeutic options.

MicroRNAs (miRNAs/miRs) are non-coding RNAs with lengths of 21–24 nucleotides that play crucial roles in the progression of OA by regulating the expression levels of their specific target genes [7]. Studies have demonstrated that miRNAs are useful for the diagnosis and treatment of OA, and the interactions between miRNA and their targets are implicated in the regulation of gene expression and homeostatic pathways in OA [8, 9]. *MiR-140* is downregulated in the cartilage of OA patients, and transfection with double-stranded *miR-140* may result in decreased matrix metalloproteinase 13 (*MMP13*) and increased type II procollagen (*COL2A1*) [10, 11]. *MiRNA-155* and *miRNA-146a* expression levels are upregulated in the later stages of OA, indicating that they may be implicated in the development and progression of OA [12]. By mediating SMAD family member 3 expression, increased *miR-16-5p* may promote the progression of OA [13]. Dysregulated *miR-9*, *miR-146*, and *miR-98* function in regulating tumor necrosis factor alpha (*TNF-α*) production in primary chondrocytes, and *miR-9* can suppress the secretion of *MMP13* in the chondrocytes of OA patients [14]. Although previous studies have found that these miRNAs and genes are involved in the progression of OA, the pathogenesis of OA is not fully understood.

In this study, the gene and miRNA expression profiles of OA were downloaded. Then, differential expression analysis was conducted to identify differentially expressed genes (DEGs) and differentially expressed miRNAs (DEMs). Furthermore, miRNA-gene prediction, functional enrichment analysis, and miRNA-transcription factor (TF)-gene regulatory network analysis were performed, and the key miRNAs and genes in OA were selected and verified. The key miRNAs and genes identified in this study may be candidate diagnostic and therapeutic targets for OA patients.

## Methods

### Data source

The OA gene expression and miRNA expression profiles were searched using the Gene Expression Omnibus (GEO) database (http://www.ncbi.nlm.nih.gov/geo/) based on the following selection criteria: (1) the dataset included blood or serum samples from OA patients and normal controls and (2) the samples did not undergo any specific treatment, such as drug treatment. Finally, the GSE105027 (miRNA expression profile, platform: GPL21575 Agilent-070156 Human_miRNA_V21.0_Microarray 046064 Feature Number version; 12 serum samples from OA patients and 12 serum samples from normal controls) and GSE48556 (mRNA expression profile, platform: GPL6947 Illumina HumanHT-12 V3.0 expression beadchip; 106 blood samples from OA patients and 33 blood samples from normal controls) datasets were selected.

### Differential expression analysis

The limma package in R (version 3.36.3; http://www.bioconductor.org/packages/release/bioc/html/limma.html) [15] was used for normalization processing. The median value of the probes corresponding to the same gene was taken as the expression value of the gene, and the average value of the probes corresponding to the same miRNA was obtained as the expression value of the miRNA. The *P* value of mRNA differential analysis was corrected for the false discovery rate (FDR), and genes with a FDR < 0.05 were regarded as DEGs. The ROCR package in R (version 1.0-7; https://cran.r-project.org/web/packages/ROCR/index.html) [16] was used to perform receiver operating characteristic (ROC) analysis of miRNAs in OA and normal samples. The area under the ROC curve (AUC) represented the ability of miRNAs to distinguish OA samples from normal samples, and a *P* value < 0.05 and |AUC-0.5| > 0.3 were set as the criteria for screening DEMs.

### Prediction of miRNA-gene pairs

The target genes of the DEMs were searched using the DIANA-TarBase (version 7.0; http://diana.imis.athena-innovation.gr/DianaTools) [17] and miTarBase (version 7.0; http://mirtarbase.mbc.nctu.edu.tw/php/index.php) [18] databases. Target genes appearing in both databases were selected as the target genes of DEMs, and the target genes that negatively correlated with DEMs were further screened [19].

### Enrichment analysis

The Gene Ontology (GO) database is valuable for predicting the biological process (BP), molecular function (MF), and cellular component (CC) functions of gene products [20]. The Kyoto Encyclopedia of Genes and Genomes (KEGG) database is typically used to predict pathways that involve certain genes [21]. Using the clusterProfiler package (version 3.8.1; http://www.bioconductor.org/packages/release/bioc/html/clusterProfiler.html) [22], the target genes that negatively correlated with DEMs underwent GO_BP and KEGG enrichment analyses. A FDR < 0.05 was defined as the significant threshold.

### Construction of the miRNA-gene network

For the DEGs that negatively correlated with DEMs, the protein-protein interaction (PPI) pairs were extracted from the STRING database (version 10.5; http://string-db.org/) [23], with a STRING score threshold of > 400. Along with the DEM-DEG pairs with negative correlations, the miRNA-gene network was constructed using the Cytoscape software (http://www.cytoscape.org) [24].

### Construction of the miRNA-TF-gene regulatory network

Using the chromatin immunoprecipitation sequencing data of the 153 TFs in the Cistrome Data Browser (http://cistrome.org/db) [25], the TFs with a FDR < 0.05 in the differential expression analysis were screened out. Then, bedtools (version 2.17.0; https://bedtools.readthedocs.io/en/latest/) [26] were used to screen the TFs that could target the genes in the miRNA-gene network. Moreover, the miRNA-TF-gene regulatory network was built using the Cytoscape software [24].

### Identification of key miRNAs and genes

The top three miRNAs targeting the highest number of genes in the miRNA-TF-gene regulatory network were considered as the key miRNAs. Regarding protein-coding genes in the miRNA-TF-gene regulatory network, searches were conducted in the National Center for Biotechnology Information PubMed database using gene name and “osteoarthritis” as keywords. Genes with search results were considered as key genes. In addition, the expression levels of key genes and the ROC curves of the key miRNAs are analyzed.

### Quantitative reverse transcription polymerase chain reaction (qRT-PCR) verification

Based on the results of the above analysis, the expression levels of the three key miRNAs and their target genes (key genes) were verified in six OA blood samples and six normal blood samples (controls). Total RNA was extracted using TRIzol reagent (No. 9109; Takara Bio, Shiga, Japan) according to the manufacturer’s instructions. The concentration and quality were determined using a microplate reader (Infinite M100 PRO; Tecan, Männedorf, Switzerland). RT of total RNA was performed using PrimeScript™RT Master Mix (No. RR036A; Takara Bio), followed by qRT-PCR using Power SYBR Green PCR Master Mix (No. A25742; Thermo Fisher Scientific, Waltham, MA, USA) under the following thermal cycling conditions: 50 °C for 2 min, 95 °C for 10 min, followed by 40 cycles at 95 °C for 10 s and 60 °C for 30 s. The melting curve was analyzed from 60 to 95 °C at an incremental rate of 0.5 °C/10 s. The primer sequences are shown in Supplementary Table 1. Finally, the relative expression levels of miRNAs and target genes were calculated according to the 2^−ΔΔCt^ method [27]. All data are presented as means ± standard deviations. Statistical analysis and graphing were performed using the GraphPad Prism 5 software (GraphPad Software, San Diego, CA, USA). *P* < 0.05 and *P* < 0.01 were used to identify results with statistically significant differences and highly significant differences, respectively.

### Ethics and consent

This study was approved by the medical ethics committee of Shanghai Fifth People’s Hospital affiliated with Fudan University, and informed consent was obtained from all patients prior to participation. All microarray datasets were published and downloaded from the GEO database; thus, we confirmed that all necessary ethical approvals and informed consent were obtained.

## Results

### Differential expression analysis

**Fig. 1:**
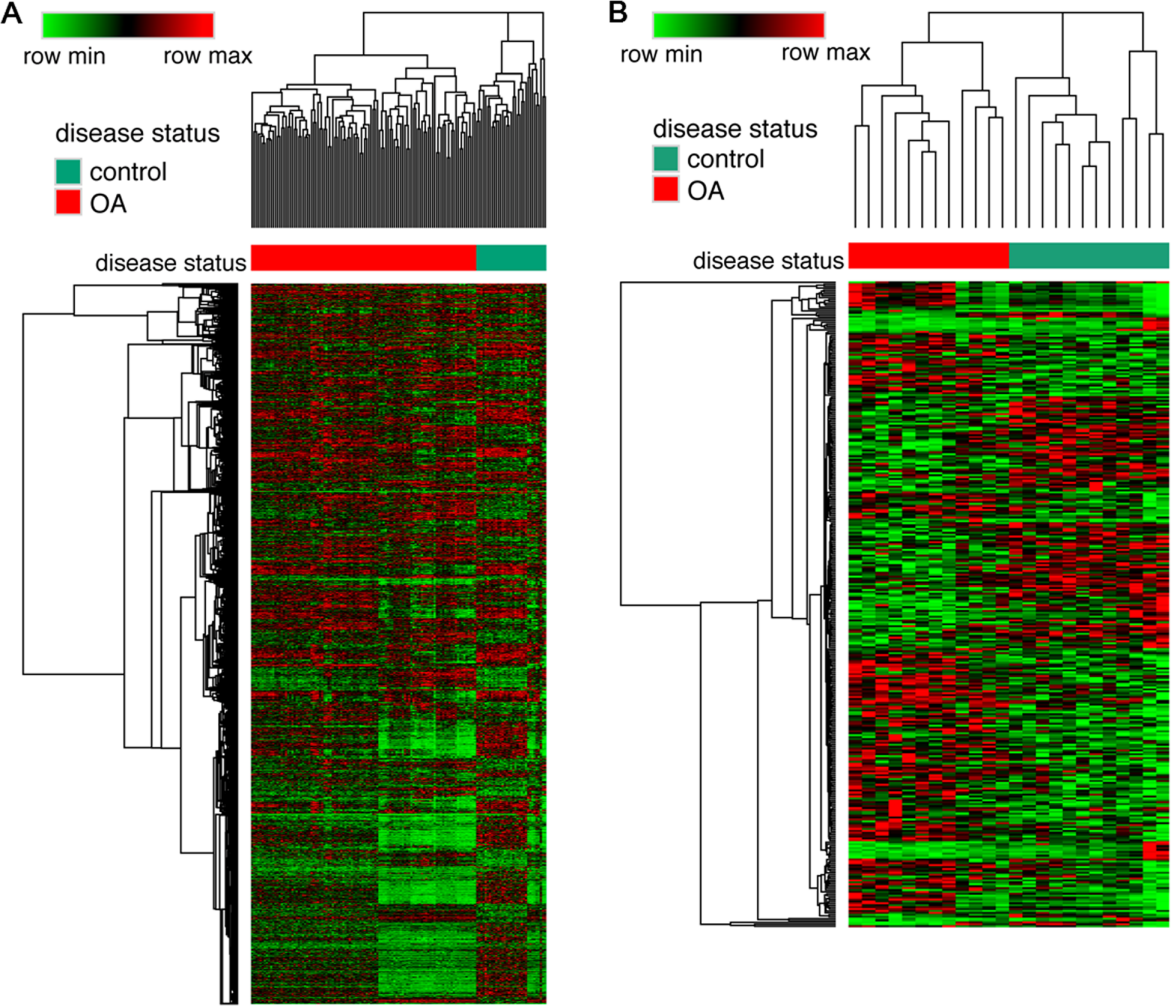
Clustering heatmaps. **a** Heatmap for the differentially expressed genes (DEGs). **b** Heatmap for the differentially expressed miRNAs (DEMs). In the sample strip, red and green represent osteoarthritis (OA) and control samples, respectively

A total of 1696 DEGs (739 upregulated and 957 downregulated) were identified between OA and normal samples (Fig. 1a). Relative to the normal samples, 108 DEMs (56 upregulated and 52 downregulated) were screened in the OA samples (Fig. 1b).

### Prediction of miRNA-gene pairs

**Fig. 2:**
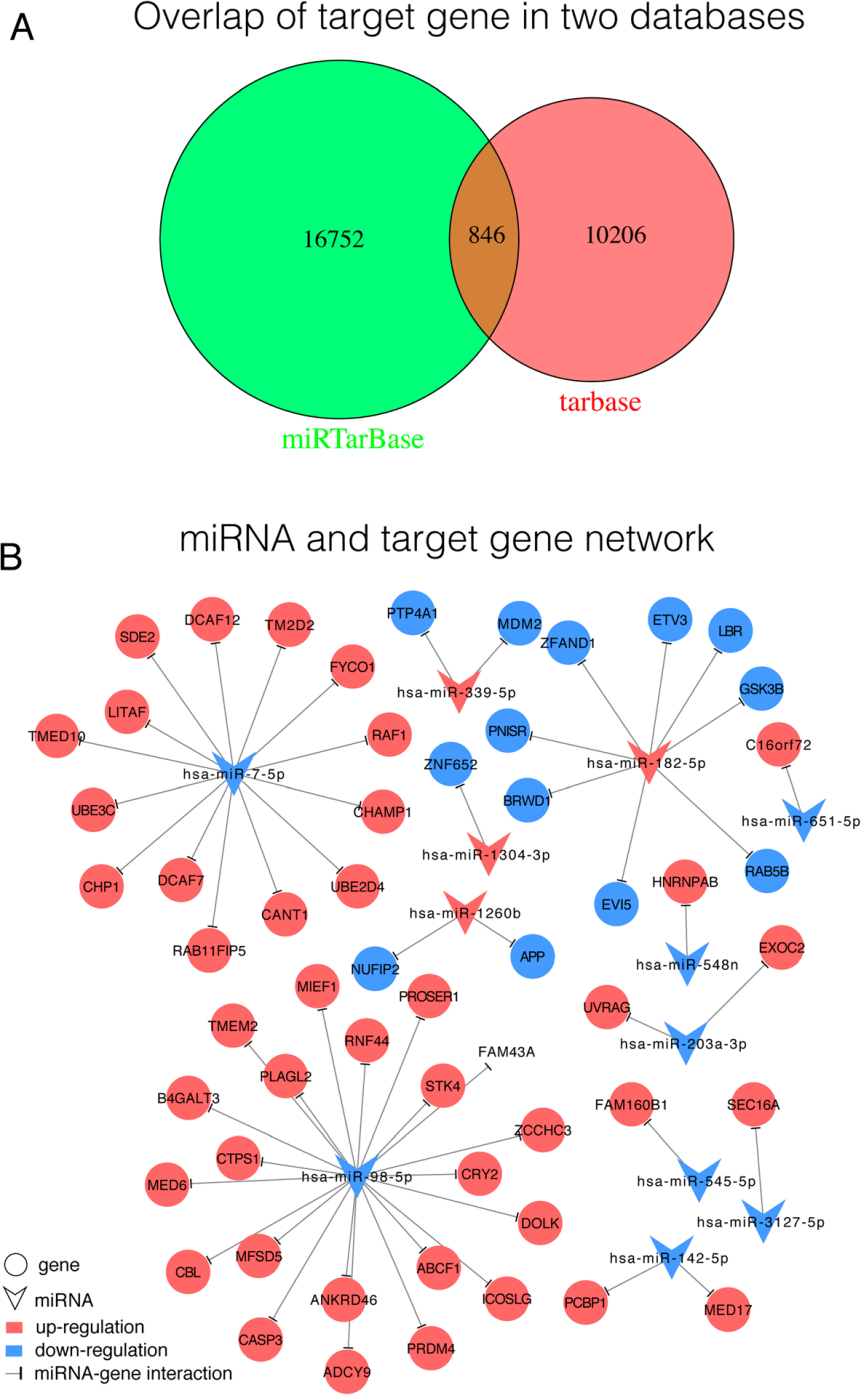
Venn diagram and negatively correlated miRNA-gene pairs. **a** Overlap of the target genes in the DIANA-TarBase (red) and miTarBase (green) databases. **b** The 56 target genes that negatively correlated with 12 miRNAs (red circles, blue circles, and inverted triangles represent upregulated genes, downregulated genes, and miRNAs, respectively). A t shape represents the miRNA-gene regulatory relationship

The 108 DEMs had 5856 (involving 11,052 miRNA-gene pairs) and 7194 (involving 17,598 miRNA-gene pairs) target genes in the DIANA-TarBase and miTarBase databases, respectively. A total of 755 target genes (involving 846 miRNA-gene pairs) appeared in both databases and thus were selected as the target genes of the DEMs (Fig. 2a). Among the 846 miRNA-gene pairs, 12 miRNAs (4 upregulated and 8 downregulated) were negatively correlated with 56 target genes (including 43 upregulated and 13 downregulated genes) (Fig. 2b).

### Enrichment analysis

**Fig. 3:**
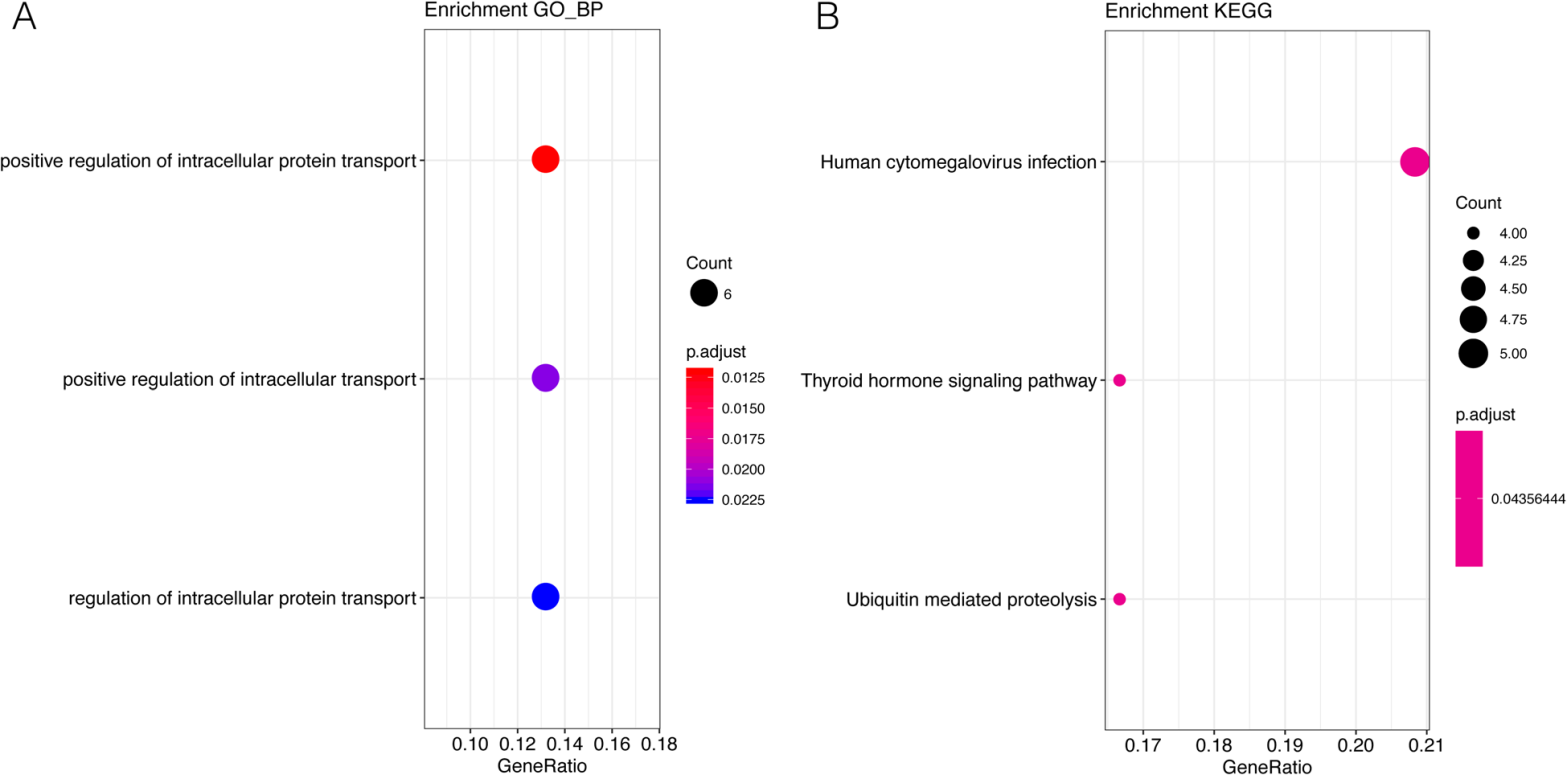
Enrichment analysis results. **a** The Gene Ontology (GO)_biological process (BP) terms enriched for the 56 target genes. **b** The Kyoto Encyclopedia of Genes and Genomes (KEGG) pathways enriched for the 56 target genes

For the 56 target genes that were negatively correlated with 12 DEMs, GO_BP and KEGG enrichment analyses were carried out. GO_BP enrichment analysis showed that three terms (including positive regulation of intracellular protein transport, positive regulation of intracellular transport, and regulation of intracellular protein transport) were enriched (Fig. 3a). Meanwhile, three KEGG pathways were enriched, including human cytomegalovirus infection, thyroid hormone signaling pathway, and ubiquitin-mediated proteolysis (Fig. 3b).

### Construction of the miRNA-gene network

**Fig. 4:**
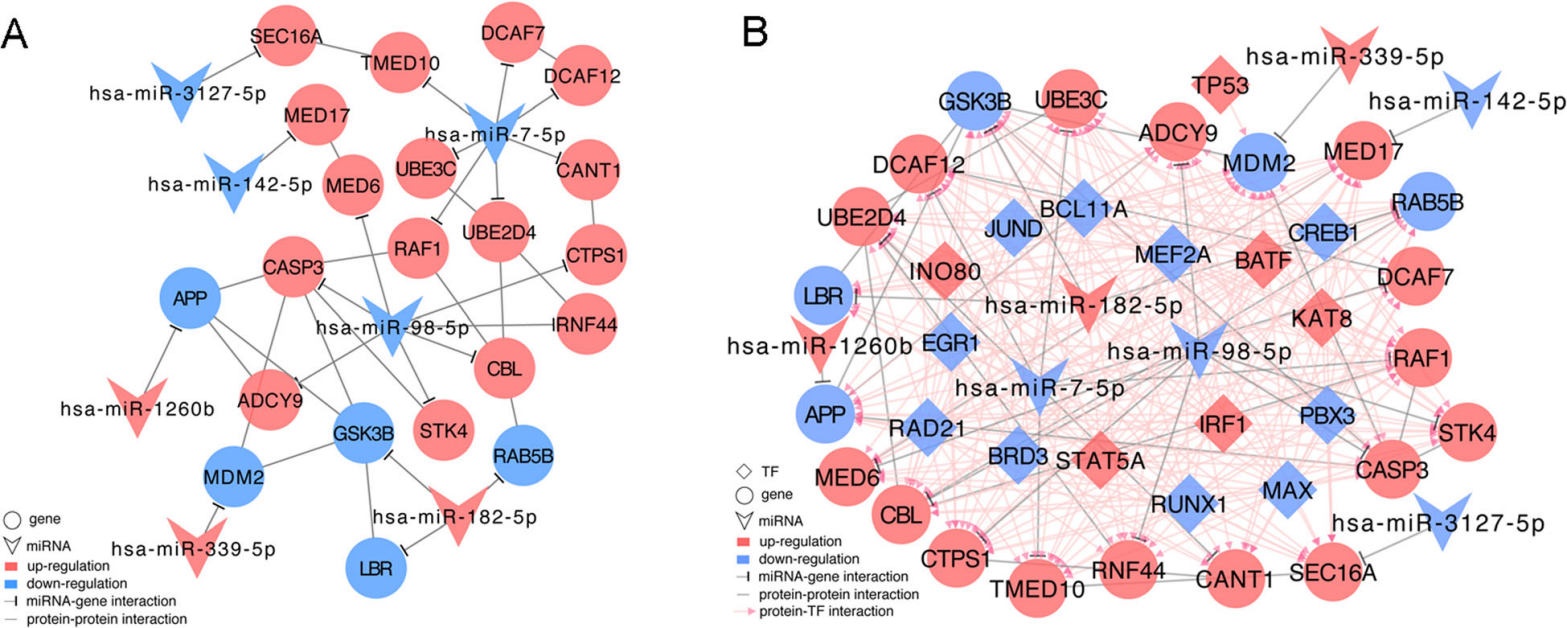
Regulatory networks. **a** The miRNA-gene network. **b** The miRNA-transcription factor (TF)-gene regulatory network. Circles, inverted triangles, and diamonds represent genes, miRNAs, and TFs, respectively. Red and blue represent upregulated and downregulated genes, respectively. T shapes, lines, and arrows represent miRNA-gene regulatory relationships, protein-protein interactions, and TF-gene regulatory relationships, respectively

After the 56 target genes were mapped into the STRING database, 21 target genes were found to be involved in the PPI pairs. In addition, the 21 target genes were targeted by 7 DEMs. Finally, the miRNA-gene network was visualized, which involved 28 nodes (21 genes and 7 miRNAs) and 39 edges (21 miRNA-gene regulatory pairs and 18 PPI pairs) (Fig. 4a).

### Construction of the miRNA-TF-gene regulatory network

A total of 17 TFs were found to be differentially expressed between the OA and normal samples, among which 16 TFs could target the genes in the miRNA-gene network. In the miRNA-TF-gene regulatory network, there were 44 nodes (21 genes, 7 miRNAs, and 16 TFs) and 280 edges (21 miRNA-gene regulatory pairs, 18 PPI pairs, and 241 TF-gene regulatory pairs) (Fig. 4b).

### Identification of key miRNAs and genes

**Fig. 5:**
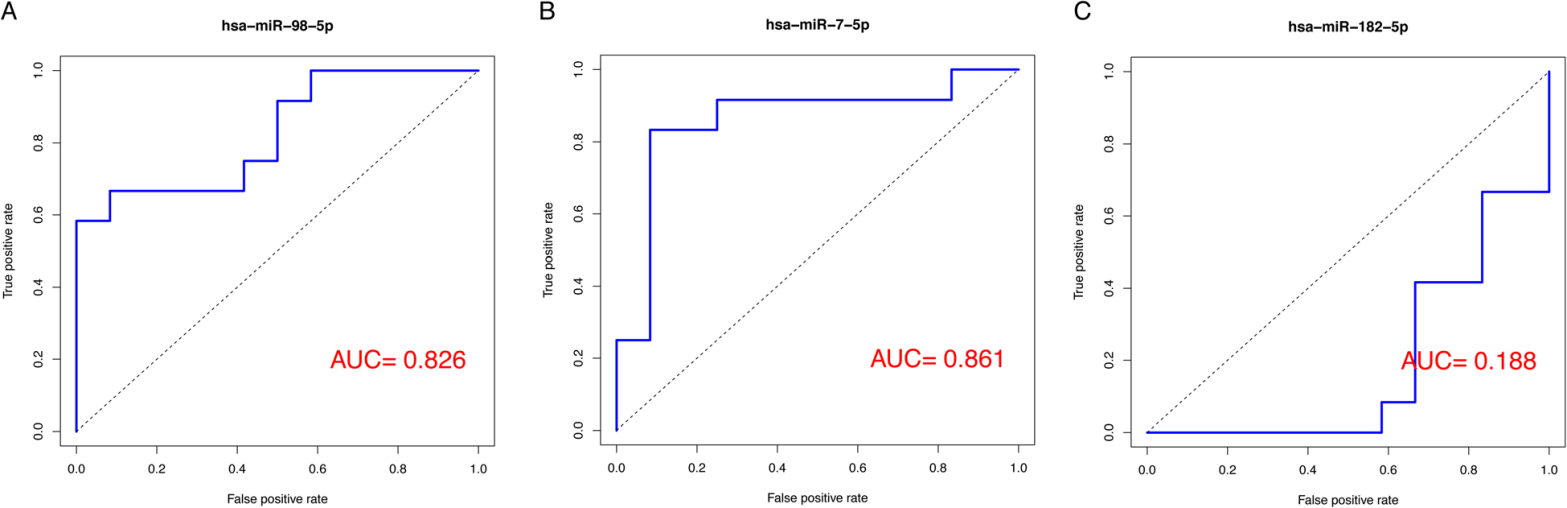
Receiver operating characteristic (ROC) curves of the three key miRNAs. **a** The ROC curve of *miR-98-5p*. **b** The ROC curve of *miR-7-5p*. **c** The ROC curve of *miR-182-5p*. AUC, area under the ROC curve

**Fig. 6:**
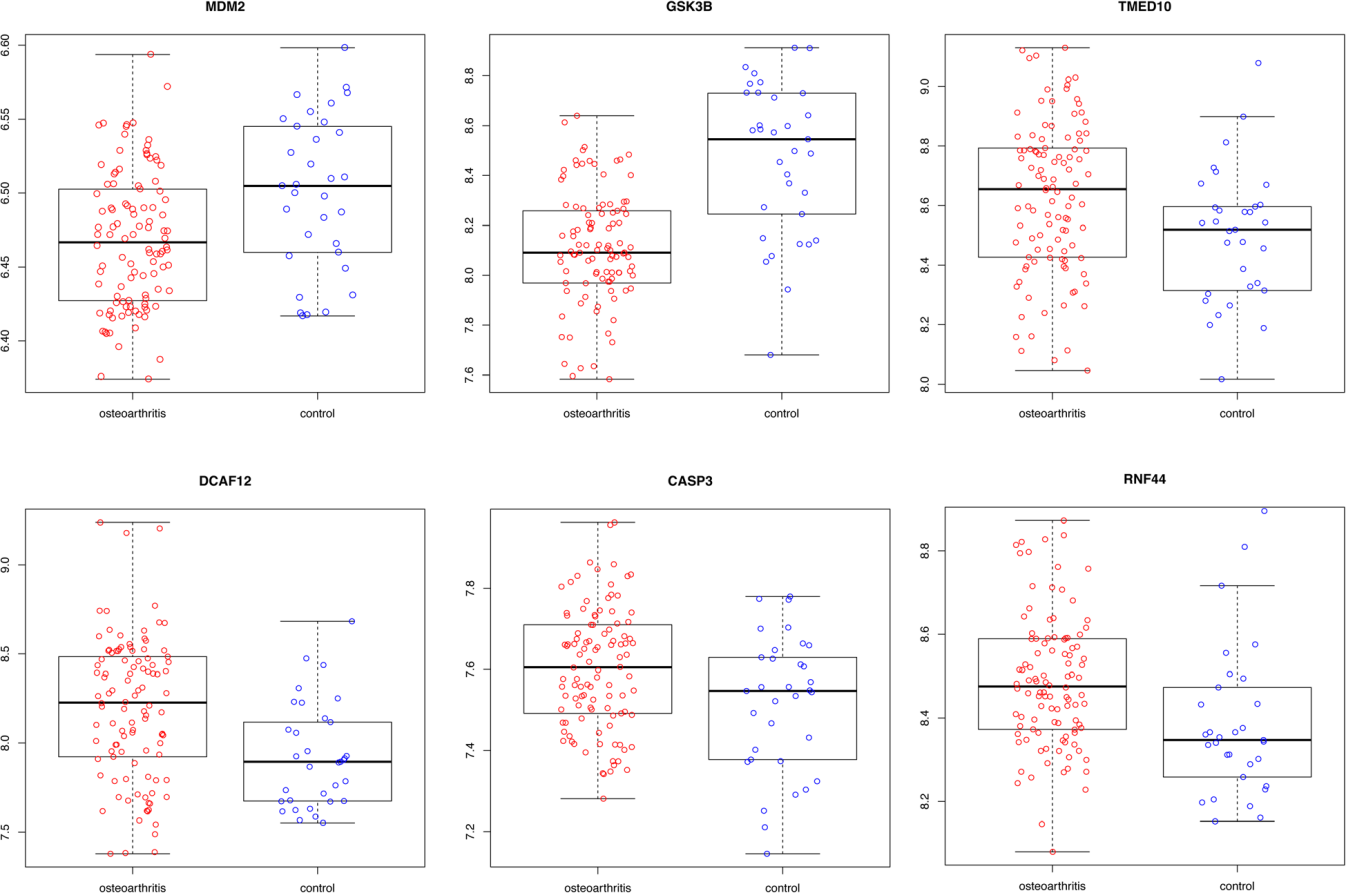
Expression levels of the six key genes (murine double minute 2, *MDM2*; glycogen synthase kinase 3-beta, *GSK3B*; transmembrane P24-trafficking protein 10, *TMED10*; DDB1 and CUL4-associated factor 12, *DCAF12*; caspase 3, *CASP3*; and ring finger protein 44, *RNF44*)

*MiR-98-5p*, *miR-7-5p*, and *miR-182-5p* were the top three miRNAs that targeted the highest number of genes in the miRNA-TF-gene regulatory network and thus were considered as the key miRNAs. Six key genes (including murine double minute 2, *MDM2*; glycogen synthase kinase 3-beta, *GSK3B*; transmembrane P24-trafficking protein 10, *TMED10*; DDB1 and CUL4-associated factor 12, *DCAF12*; caspase 3, *CASP3*; and ring finger protein 44, *RNF44*) had search results in the PubMed database. The ROC curves of the three key miRNAs and the expression levels of the six key genes are shown in Figs. 5 and 6, respectively. In particular, the *miR-7-5p → TMED10/DCAF12*, *miR-98-5p → CASP3/RNF44*, and *miR-182-5p → GSK3B* regulatory pairs as well as the *MDM2─GSK3B/CASP3* PPI pairs were present in the miRNA-TF-gene regulatory network.

### Expression levels of the key miRNAs and genes

The expression levels of the three key miRNAs (*miR-98-5p*, *miR-7-5p*, and *miR-182-5p*) and three key target genes (*TMED10*, *CASP3*, and *GSK3B*) were verified using qRT-PCR. As shown in Supplementary Figure 1, the OA samples displayed a significant decrease in the expression levels of *miR-7-5p* and *miR-98-5p* and a significant increase in the expression level of *miR-182-5p* compared to those of the control samples. In addition, the expression levels of *TMED10*, *CASP3*, and *GSK3B* in the OA samples changed as expected. More specifically, the *TMED10* (the target gene of *miR-7-5p*) and *CASP3* (the target gene of *miR-98-5p*) expression levels were significantly upregulated while that of *GSK3B* (the target gene of *miR-182-5p*) was significantly downregulated in the OA samples compared to the controls.

## Discussion

In this study, 1696 DEGs (739 upregulated and 957 downregulated) and 108 DEMs (56 upregulated and 52 downregulated) were screened in OA samples relative to normal samples. Among the predicted miRNA-gene pairs, 56 target genes were negatively correlated with 12 DEMs. For the 56 target genes, three GO_BP terms (such as positive regulation of intracellular protein transport) and three KEGG pathways (such as human cytomegalovirus infection) were enriched. In addition, the miRNA-gene and miRNA-TF-gene regulatory networks were constructed. Moreover, three key miRNAs (*miR-98-5p*, *miR-7-5p*, and *miR-182-5p*) and six key genes (*MDM2*, *GSK3B*, *TMED10*, *DCAF12*, *CASP3*, and *RNF44*) were screened, among which the miR-7-5p → TMED10/DCAF12, miR-98-5p → CASP3/RNF44, and miR-182-5p → GSK3B regulatory pairs were found to be present in the regulatory network. In particular, the expression levels of *TMED10* and *miR-7-5p*, *CASP3*, *miR-98-5p*, *GSK3B*, and *miR-182-5p* showed a negative correlation with qRT-PCR verification.

*MiR-7-5p* and *miR-214-5p* expression levels are elevated in rheumatoid arthritis (RA) with interstitial lung disease (ILD), which may serve as diagnostic markers for RA-associated ILD [28]. *TMED10* expression is critical for transforming growth factor β signaling [29], which influences cartilage integrity as well as the prevention and repair of cartilage damage [30]. *DCAF12* belongs to the WD40-motif repeat protein family, which has evolutionary conservation and exerts multiple roles in signal transduction [31]. The *miR-7-5p → TMED10/DCAF12* regulatory pairs existed in the miRNA-TF-gene regulatory network, indicating that *miR-7-5p* may act by regulating *TMED10* and *DCAF12* in OA.

*MiR-98* expression is decreased in OA chondrocytes, and its inhibition can result in chondrocyte apoptosis; therefore, *miR-98* may be applied in targeted OA therapy [32, 33]. By enhancing extracellular signal-regulated kinase expression and inhibiting *CASP3*, *FAS*, and Fas ligand expression, recombinant human lactoferrin can reverse dexamethasone-induced chondrocyte apoptosis in OA [34]. Upregulated Piezo1 affects the apoptotic rate of OA chondrocytes via a *CASP12*-dependent pathway [35]. Through the histone deacetylase 9 protein inhibitor of the activated STAT Y-*RNF4* axis, the post-translational modification of *Nkx3.2* acts to mediate chondrocyte hypertrophy during the course of skeletal development [36]. *RNF4* is targeted by Epstein-Barr virus *miR-BHRF1-1*, and its reconstitution is related to decreased viral proteins and damaged virus release [37]. The *MiR-98-5p → CASP3/RNF44* regulatory pairs were involved in the miRNA-TF-gene regulatory network, suggesting that *miR-98-5p* may affect the pathogenesis of OA by targeting *CASP3* and *RNF44*.

*MiR-182* positively mediates T helper cell proliferation by suppressing forkhead box O1 expression, and its inhibition prevents T helper cells from inducing arthritis [38]. *GSK3B* maintains the chondrocytic phenotype and cartilage extracellular matrix by suppressing β-catenin, and its inhibition promotes the differentiation of OA chondrocytes [39]. The *MiR-182-5p → GSK3B* regulatory pair was found in the miRNA-TF-gene regulatory network; therefore, *miR-182-5p* may also be implicated in the development of OA by mediating *GSK3B*. *MDM2* expression in the fibroblast-like synoviocytes of RA patients was upregulated compared to that of OA patients, which may help to reduce apoptosis of lining tissues in RA [40]. *MDM2* interacted with *GSK3B* and *CASP3* in the miRNA-TF-gene regulatory network; thus, *MDM2* may function in the OA mechanisms via interaction with *GSK3B* and *CASP3*.

Despite these findings, this study has some limitations. The lack of human disease tissues that reflect disease characteristics limits the microarray study of human diseases, including OA. Considering the roles of peripheral blood in immune response, metabolism, and intercellular communication, especially the simplicity of sample collection, it has been considered as a notable tissue for biomarker detection. Therefore, a microarray dataset of blood samples was selected and analyzed. However, blood samples could be affected by many factors, and the data from tissue samples might provide more reliable results. This is one limitation of the present study. In addition, we only confirmed the mRNA expression levels of the key genes and miRNAs. MiRNA-mRNA interactions should be further investigated using a dual-luciferase reporter assay. Moreover, the protein expression levels of key target genes should be further confirmed by western blot analysis. Finally, the roles of these key genes and miRNAs in the development and progression of OA should be investigated using functional experiments.

In conclusion, 1696 DEGs and 108 DEMs were identified between OA and normal samples. Additionally, *miR-98-5p*, *miR-7-5p*, *miR-182-5p*, *MDM2*, *GSK3B*, *TMED10*, *DCAF12*, *CASP3*, and *RNF44* may play important roles in the pathogenesis of OA. However, the functions of these key miRNAs and genes in OA need to be validated by future experiments.

## Supplementary Information

**Additional file 1: Supplementary Table 1.** Primer sequences of key miRNAs and genes used in qRT-PCR.

**Additional file 2: Supplementary Figure 1.** Expression levels of three key miRNAs and three key target genes determined by qRT-PCR. *MiR-98-5p* and *miR-7-5p* were downregulated while *miR-182-5p* was upregulated in OA samples compared to the controls (*P* < 0.01). *MDM2* and *CASP3* were upregulated while *GSK3B* was downregulated in OA samples compared to the controls (*P* < 0.01).

## Data Availability

The datasets used and analyzed during the current study are available from the corresponding author upon reasonable request.

## Abbreviations

BP: Biological process
CC: Cellular component
DEGs: Differentially expressed genes
DEMs: Differentially expressed miRNAs
GEO: Gene Expression Omnibus
GO: Gene Ontology
KEGG: Kyoto Encyclopedia of Genes and Genomes
MF: Molecular function
OA: Osteoarthritis
RA: Rheumatoid arthritis
TF: Transcription factor
