# Supplementary material for: Bioinformatics analysis of miRNA and mRNA expression profiles to reveal the key miRNAs and genes in osteoarthritis
Source: J Orthop Surg Res. 2021 Jan 19;16:63. doi: 10.1186/s13018-021-02201-2 (PMC7814623; doi:10.1186/s13018-021-02201-2)
Supplement: Supplementary file 1 — Additional file 1: Supplementary Table 1. Primer sequences of key miRNAs and genes used in qRT-PCR. [file 13018_2021_2201_MOESM1_ESM.doc]

**Supplementary table 1.** The primer sequences of key miRNAs and genes in RT-qPCR.

| Name | Primer sequences |
| --- | --- |
| hsa-miR-7-5p-RT | GTCGTATCCAGTGCAGGGTCCGAGGTATTCGCACTGGATACGACAACAAC |
| JH-miR-7-5p-F | GCGCTGGAAGACTAGTGATTTT |
| hsa-miR-98-5p-RT | GTCGTATCCAGTGCAGGGTCCGAGGTATTCGCACTGGATACGACAACAAT |
| JH-miR-98-5p-F | GCCGCTGAGGTAGTAAGTTGT |
| hsa-miR-182-5p-RT | GTCGTATCCAGTGCAGGGTCCGAGGTATTCGCACTGGATACGACAGTGTG |
| JH-miR-182-5p-F | GCGCTTTGGCAATGGTAGAACT |
| JH-R | GTGCAGGGTCCGAGGT |
| TMED10-hF | GGTGGTGAACGAGTCTCCAG |
| TMED10-hR | AAAAGCAGCAACGCTAACGG |
| CASP3-hF | CGGCGCTCTGGTTTTCGTTA |
| CASP3-hR | GAGGTTTGCTGCATCGACAT |
| GSK3B-hF | AGGATTCGTCAGGAACAGGACA |
| GSK3B-hR | TTGAATCCGAGCATGAGGAGG |
| GAPDH-hF | TGACAACTTTGGTATCGTGGAAGG |
| GAPDH-hR | AGGCAGGGATGATGTTCTGGAGAG |
| U6-hF | CTCGCTTCGGCAGCACA |
| U6-hR | AACGCTTCACGAATTTGCGT |
