# Supplementary figures and images for: Bioinformatics analysis of miRNA and mRNA expression profiles to reveal the key miRNAs and genes in osteoarthritis
Source: J Orthop Surg Res. 2021 Jan 19;16:63. doi: 10.1186/s13018-021-02201-2 (PMC7814623; doi:10.1186/s13018-021-02201-2)

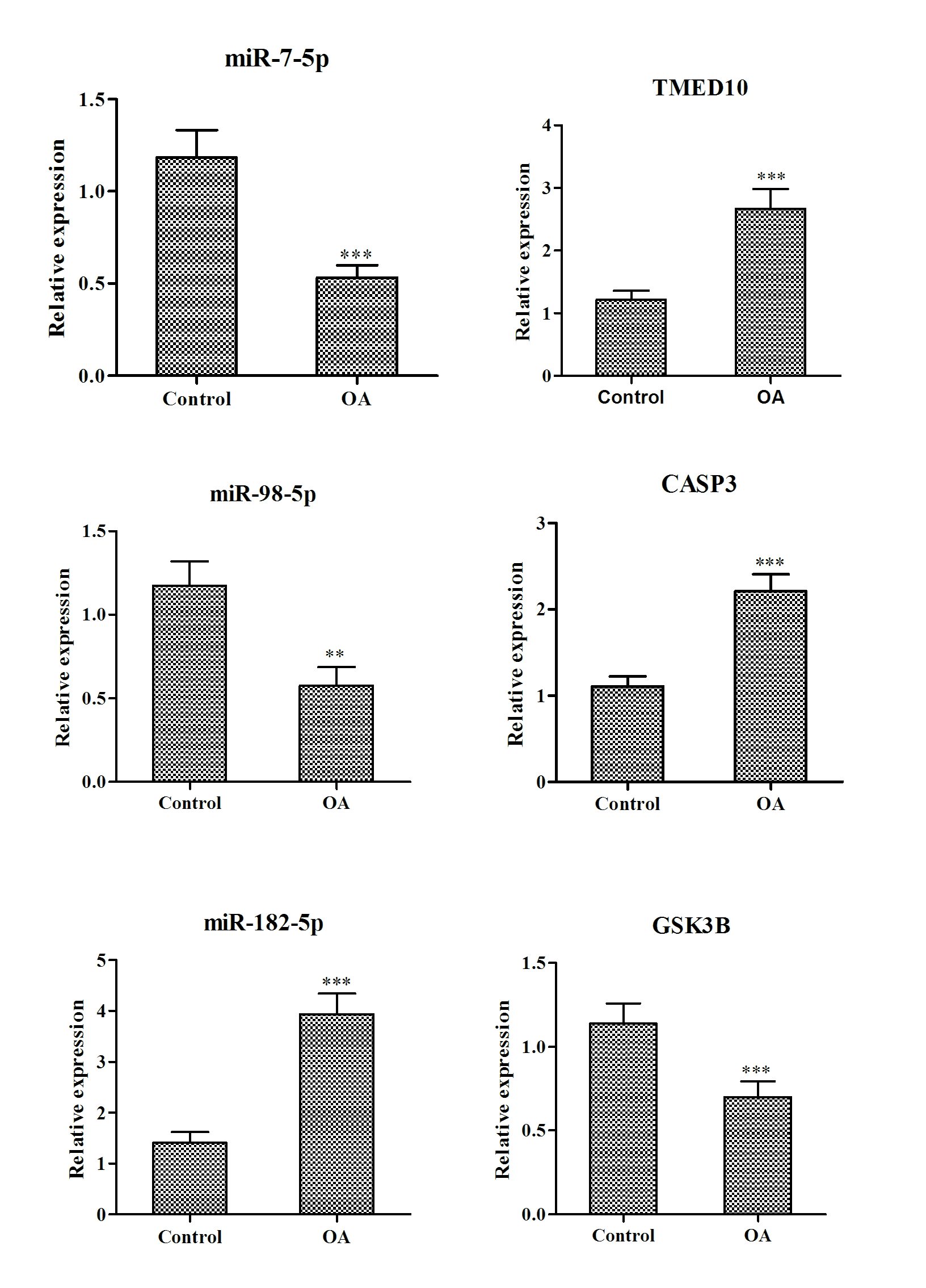

Supplement: Supplementary file 2 — Additional file 2: Supplementary Figure 1. Expression levels of three key miRNAs and three key target genes determined by qRT-PCR. MiR-98-5p and miR-7-5p were downregulated while miR-182-5p was upregulated in OA samples compared to the controls (P < 0.01). MDM2 and CASP3 were upregulated while GSK3B was downregulated in OA samples compared to the controls (P < 0.01). [file 13018_2021_2201_MOESM2_ESM.tif]
